# Access to advanced cancer care services in the West Bank-occupied Palestinian territory

**DOI:** 10.3389/fonc.2023.1120783

**Published:** 2023-03-16

**Authors:** Suzan Mitwalli, Weeam Hammoudeh, Rita Giacaman, Richard Harding

**Affiliations:** ^1^ Institute of Community and Public Health, Birzeit University, West Bank, Palestine; ^2^ Florence Nightingale Faculty of Nursing, Midwifery and Palliative Care, Cicely Saunders Institute, London, United Kingdom

**Keywords:** occupied Palestinian territory (oPt), access, cancer, care, services, Israeli military occupation; conflict, challenges

## Abstract

**Introduction:**

Universal Health Coverage (UHC) identifies the provision of palliative care for people with advanced disease as an essential health service. Palliative care is also stipulated as a human right under existing covenants. Oncology services provided by the Palestinian Authority under Israeli military occupation are limited to surgery and chemotherapy treatment. Our study aimed to describe the experiences of patients with advanced-stage cancer in the West Bank in accessing oncology services and meeting their health care needs.

**Methodology:**

We conducted a qualitative study among adult patients diagnosed with advanced lung, colon, or breast cancer in three Palestinian governmental hospitals, and with oncologists. Thematic analysis was conducted on the verbatim interview transcripts.

**Results:**

The sample consisted of 22 Palestinian patients (10 men and 12 women) and 3 practicing oncologists. The findings reveal that cancer care is fragmented, with limited access to the services needed. Patients face referral delays in accessing treatment which worsen their health condition in some cases. Some patients reported difficulties in getting Israeli permits to access radiotherapy treatment in East-Jerusalem, and others experienced interruptions of their chemotherapy treatment sessions due to the unavailability of chemotherapy medications caused by Israeli-side delays. Other reported problems were related to the Palestinian health system and service delivery and quality, including fragmentation of services, infrastructure issues, and unavailability of medications. Advanced diagnostic services and palliative care are almost absent at Palestinian governmental hospitals, and patients need to seek these services in the private sector.

**Conclusion:**

The data demonstrate specific access restrictions to cancer care in the West Bank due to Israeli military occupation of Palestinian land. This affects all stages of the care pathway, from restricted diagnosis services, to limited treatment and then poor availability of palliative care. Cancer patients will continue to suffer if the root causes of these structural constraints are not addressed.

## Introduction

Cancer is a leading cause of death in low-and middle-income countries (LMIC), where higher rates of cancer mortality exist compared to high-income countries (1–3). This can partially be explained by a higher proportion of cancer patients diagnosed at late stages and lack of access to the needed health services (2, 4). Additionally, it is expected that by year 2035, the incidence rate of cancer will rise in LMIC, with a projection that two-thirds of cancer cases will be present in developing countries, including countries in the Middle East and North Africa (MENA) (2, 5).

Developing countries face many challenges in providing cancer care (1–5), with health systems incapable and unprepared to provide comprehensive cancer care. Cancer control, including prevention, screening and early diagnosis, is hardly available and is inaccessible for many people (3). Moreover, cancer treatment is constrained by a lack of advanced diagnostic services, a dearth of oncology health providers, shortage of radiation therapy services, and the fragmentation of cancer services (2, 3). In areas affected by armed conflict, as in some MENA countries, cancer care is much more difficult to obtain (5, 6). Physicians’ departure “brain drain”, is an additional challenge (7, 8). By 2060, serious health-related suffering will be experienced by 16.3 million people dying with cancer each year (compared to 7.8 million in 2016). The increase will be greatest in low- and middle-income countries (9–11).

In the Israeli-occupied Palestinian territory (oPt), the focus of this study, cancer was the second leading cause of death in 2017 and 2019 (12, 13). Similar to other conflict-affected areas, access to cancer care in the oPt is hampered by numerous impediments. In this paper, we aim to understand experiences of patients with advanced cancer in accessing care and their care needs in the West Bank, including Palestinian East-Jerusalem, which is illegally annexed by Israel and not accessible to Palestinians from the West Bank without permits (which are difficult to obtain).

The oPt consists of the West Bank, Gaza Strip and Palestinian East Jerusalem (14). East Jerusalem has been controlled by Israel since its annexation in 1967 and is isolated from the rest of the West Bank by the Israeli Separation Wall (15). Moreover, the West Bank is severely fragmented due to the building of illegal Israeli settlements, the Separation Wall, and the Israeli permanent and flying military checkpoints. Movement of people and goods is restricted, with ongoing direct and indirect violence against Palestinians (14), detentions, demolition of homes and attacks on Palestinian land (15). This complexity creates significant barriers to accessing health care (16–18).

Cancer treatment in the West Bank is mainly provided in small oncology units in three Palestinian governmental hospitals in addition to newly established oncology units in two private hospitals that are contracted by the Palestinian Ministry of Health (MoH) (12, 13). The governmental oncology units lack specialized pathology laboratories, and advanced diagnostic and imaging facilities. They mainly offer chemotherapy treatment and some surgical care. Moreover, there is no radiation therapy in any of the West Bank hospitals (these are not permitted by Israeli authorities) and patients who need radiotherapy are usually referred to East Jerusalem or hospitals in Israel (12, 13, 19). Referrals for cancer patients to East Jerusalem, Israel and neighboring countries are also provided for advanced diagnostic services such as nuclear medicine scanning and advanced treatment (12, 19, 20).

To access cancer services that are not available in hospitals within Palestinian Authority areas in the West Bank and Gaza Strip, cancer patients from West Bank and Gaza Strip need special Israeli permits to access these services at an East-Jerusalem Palestinian-led hospital with comprehensive oncology services (12, 13, 20). The process for obtaining Israeli permits is lengthy, confusing, and entails a lot of uncertainty (17, 20, 21). Of all permit applications from the Gaza Strip to attend hospital appointment in September 2021, 69% were accepted and 30% had no response by the appointment date. In the West Bank for the same period, 84% were approved, 12% were denied and 4% were waiting for a reply (22). Cancer patients with permit applications initially denied or delayed are less likely to survive (20).

Companions for patients attending clinical appointments/admissions are crucial to provide social support (especially for children and older people) and to provide informal care due to staff resource limitations. However, only 39% of the permit requests for companions from the Gaza Strip in September 2022 were approved, while 59% received no response. In the West Bank, 78% of companion applications were approved, 17% were refused, and 5% received no response (22).

Given the urgent need to meet the palliative care goals of UHC, evidence is needed to understand the pathway to advanced cancer care that can inform health system improvement. Our study aims to describe the experiences of patients with advanced-stage cancer in the West Bank in accessing oncology services and meeting their health care needs.

## Materials and methods

This study utilizes a qualitative methodology with in-depth interviews conducted between September-November 2021.

### Setting

The study was conducted in three Palestinian governmental hospitals located in the north, center and south of the West Bank.

### Ethics

Ethical approval was granted by the Research Ethics Committee at the Institute of Community and Public Health, Birzeit University (ref number 2020(3 – 1) and at Kings College London (ref number HR-20/21-18199).

### Recruitment and data collection

Inclusion criteria for potential participants (identified by medical oncologists working in the three participating hospitals) were as follows: adults with advanced-stage cancer (stage 3 or 4, as determined by their treating oncologist) of the lung, colon, or breast. These primary cancers were selected as they had the highest mortality rates in the West Bank in 2019 (13). We purposively sampled patients based on locality, gender, age and type of cancer care they receive. We informed the medical oncologists working in the three hospitals of our inclusion criteria. They selected participants from the hospitals' registered patients based on the criteria who were present on the day of our visit to the hospital for us to approach. We made sure to have variety in the selected participants with attention to cancer type and gender. Exclusion criteria were: patients having any other type of cancer or cancer stage 1 or 2 (as determined by their treating oncologist), or patients who were unaware of their cancer diagnosis. Selected patients were approached and briefed by the study researchers on the purpose and goals of the study, and provided with information sheets. Informed consent was obtained orally from participants, including consent to record the interviews. Based on our research experience and understanding of the local context, we have found that people become uneasy when asked to sign consent and generally prefer oral consent. Interviews lasted between 30 and 90 minutes and were conducted by researchers from the Institute of Community and Public Health (Birzeit University).

The semi-structured topic guide included diagnosis, treatment plan, knowledge about disease and treatment, coping strategies, effects on the patient’s life, the role of the family, pain management, challenges in accessing cancer treatment, communication with health providers, support accessed, and patients’ recommendations to improve cancer care.

Additionally, we interviewed oncologists working in the hospitals where we recruited the patients, to better understand the treatment protocols, referral system, and cancer services provided at these hospitals. The primary aim of the interviews with oncologists were to understand the context of care. The interviews with the oncologists were conducted before the patients' interviews and were not meant to explain identified themes resulting from patients' interviews. They were selected for the interviews because they are the main oncologists in the participating hospitals, and they have extensive knowledge of the cancer services in the West Bank. The appointments for the interviews with the oncologists were arranged through preliminary visits or phone calls. The oncologists provided oral consent to conduct the interview but did not consent to being recorded. Consequently, the researcher(s) took written notes during the interview. The notes were saved on a password-protected computer. All interviews with patients were audio recorded, transcribed verbatim, and files saved on a password-protected computer.

### Data analysis

We analyzed the data using a thematic analysis approach (23). We first coded the interview transcriptions and notes, and the codes were then categorized into themes. Afterwards, we arranged themes into a thematic network, which we explored and described, and then summarized, and finally produced interpretative patterns. To strengthen the analysis, we also created analytical memos (24). Quotes were extracted from transcriptions and notes and translated into English. We then structured the results section based on the thematic analysis, analytical memos and our research questions. Each theme is illustrated using verbatim quotes with a participant ID and brief description to demonstrate the breadth of sample.

## Results

### Sample characteristics

We recruited and interviewed 22 patients in total (10 men and 12 women), with an age range of 30-71 years. Nine patients came from the north, three from the center, and ten from the south of the West Bank. Fourteen people had stage 4 cancer, and eight people had stage 3 cancer. Time since diagnosis ranged from one week to 14 years. Five interviews were conducted in an inpatient ward, while the rest were with patients attending daycare. Moreover, we interviewed three lead oncologists in the governmental hospitals that have oncology units located in the north, center and south of the West Bank (assigned to us by the Ministry of Health).

### Main findings

The analysis identified three main themes: 1) Obstacles to cancer care attributable to Israeli military occupation; 2) Health system challenges; 3) Service delivery and quality.

#### Obstacles to cancer care attributable to military occupation

One of the main obstacles to cancer care is obtaining Israeli permits to enter Palestinian East-Jerusalem, particularly for radiotherapy treatment. This service is only available in the Palestinian-led hospital in East-Jerusalem, with Palestinian health care centers on the West Bank not allowed to obtain or operate such treatment by Israel. Additionally, some patients seek advanced diagnostic services in East-Jerusalem that are not available in Palestinian governmental hospitals, such as nuclear scans. Participants’ complaints about Israeli permits included delays in obtaining them or refusal, for instance, because of so-called “security” related reasons:

“*… I do not receive permits from a security point of view* (i.e. denied on the basis of ‘security’ by the Israeli military). *I tried before and it did not work….I tried to apply for a permit from the Israelis to go to “A” hospital* (the Palestinian led East-Jerusalem hospital) *for a checkup, the Israeli authorities did not agree, the Israeli interrogator interrogated me and told me I am not allowed*.”

(Interview 11, male, 58 years old, rural, north WB)

Another participant shared his experience of being unable to get the permit usually given to patients because he already had what is called a commercial permit which does not allow him to cross the usual East-Jerusalem crossing. Instead, he has to go another way. This prolongs his travel to East-Jerusalem and separates him from his son, who must pass through the usual East-Jerusalem crossing alone.

“*By God, these permits are burdensome and taxing, burdensome and taxing…. Even though I have a commercial permit, they issued a permit for my son and they did not issue me a permit, not allowed. (With) my commercial permit, (I have to) go into the tunnel (a different crossing from his son), they said let the boy in and I was not allowed. The Israeli soldier at the checkpoint said: you should go down and around to Bethlehem (in the south) to the checkpoint there, and the Israeli army checkpoint was so congested with workers (Palestinian workers wanting to cross to work in Israel) and I should have been there with my son at 7.30 am.”*

(Interview 7, male, 54 years old, rural, south WB)

Some participants with a Jerusalem identity card (ID), which is considered by Israel an Israeli ID and allows free passage between the West Bank and Israel, including East-Jerusalem reported being unable to access care in East-Jerusalem. A Palestinian woman who has a Jerusalem ID and is married to a man with a West Bank ID had her Israeli health insurance terminated as she now resides in the West Bank.

*“My health insurance was cut [stopped]. If I want to go and renew my health insurance, it would take me months and I do not know what will happen to my health state.”*

(Interview 3, female, 38 years old, urban, south WB)

Another patient, who lives in Bethlehem in the south of the West Bank but has a Jerusalem ID, preferred to access treatment in a Palestinian governmental hospital because she was concerned about the travel time and high transportation costs to reach treatment centers in Jerusalem.

*“They get you tired when you have to go out at 6 am and reach Jerusalem at ten and pay 100 Israeli shekels for transport, and you want food, and you want drink, and you get tired before you sit on the chair for Israeli hospital treatment. Here in fifteen minutes, I reach the hospital and the doctors.”*

(Interview 8, female, 50 years old, rural, south WB)

Patients also reported interruptions in accessing diagnostic and treatment services due to Israeli delays in providing supplies to the Palestinian hospitals, such as medical materials needed for nuclear medicine in addition to the chemotherapy drugs.

“*Now I have to get a nuclear image/scan in Hebron, but the material did not arrive from Israel.”*

(Interview 8, female, 50 years old, rural, south WB)

*“There are some days when the medication (chemotherapy) is there and at other days the medications are not there so they are obliged to postpone giving medications to patients until medications arrive. We also faced this because this medication comes from Israel, and Israel gives them the medication according to the number of patients they have. It does not give them the medication in large quantities, only based on the number of patients who need it, meaning if they have 800 patients, so only 800 doses of medication.”*

(Interview 10, male, 48 years old, urban, south WB)

*“Last week, I didn’t get my chemotherapy treatment. I came, and they (at the hospital) told me that there are no medications, so they postponed my session for two days and gave me two doses to make up for it.”*

(Interview 18, female, 46 years old, rural, north WB)

#### Health system challenges

In the following sections, we discuss the Palestinian health system challenges and service delivery and quality.

##### Diagnostic services

One of the main problems of cancer care on the West Bank is the lack of advanced diagnostic services at Palestinian governmental hospitals. Some patients reported that even when the diagnostic services are available in governmental hospitals, they are sometimes not functioning, or it takes a long time to make an appointment or to get the scan report. Moreover, nuclear scans are offered only in the one hospital in East-Jerusalem and one governmental hospital in Hebron in addition to a few private hospitals.

*“If we want to send a message to someone or to those responsible in the governmental hospital, there must be MRIs and CT scans operational, and not having to come to do these tests and they tell you today we cannot because the machine is out of order…if a machine is out of order in a government hospital which serves all of Bethlehem and Hebron and there is only one machine?! A hospital with an oncology unit and you cannot get a nuclear scan done! You have to go to either “B” hospital or “A” hospital…A hospital with an oncology unit does not have a colored CT scan…I came to “C” hospital to do a CT, I may do it today and after week or two weeks I will get the scan report.”*

(Interview 5, male, 33 years old, rural, south WB)

This frustration with waiting to receive care, especially scans, was described by another participant:

*“In this hospital, if you want a scan, you have to wait a month to have your turn if you do not have wasta (connection)… radiation yes, it is available but you go and find that the machine is out of order, you may need one month, two months to be able to have the image.”*

(Interview 6, male, 45 years old, rural, south WB)

Due to the shortage of advanced diagnostic services in Palestinian governmental oncology departments, patients are referred to other governmental hospitals or the Augusta Victoria hospital in East-Jerusalem or private hospitals that are contracted by the Ministry of Health to receive these services. Participants who must go through a bureaucratic, lengthy, referral process view delays in receiving treatment as disease progresses, in turn causing additional strain and frustration.

*“From March till August, I was going from a physician to another till they do the CT scan for me…then we knew in October when they took the biopsy that I am in stage 4.”*

(Interview 12, male, 57 years old, urban, north WB)

At times, due to referral delays, some patients choose to pay for diagnostic services in private hospitals rather than going through the lengthy referral process. It is important to note that for many patients, this was a significant expense, albeit one that they viewed as necessary in order to improve their treatment outcomes.

*“. Almost all the tests are at my expense… for example the doctor today told me I must do a test using my insurance, but I would have to wait two or three days, so I am obliged to do it at my expense.”*

(Interview 9, female, 65 years old, urban, south WB)

In addition to delays, other patients reported problems which were related to ineffective or wrong diagnoses. At times discrepancies in advice or diagnoses would push patients and their families to seek care in other hospitals, often at their own expense. For some, it also affected their confidence in the care that was provided to them and for many, it was a source of stress. Below, we can see the experiences of two men.

*“We went to the hospital emergency department, and a surgeon came to us and said that these tests indicate that I do not have anything. But how nothing? I have something. The doctor said you do not have anything…. He told me throw away this medication and that medication, and of course he wrote me other medications. My son began to scream and said no, my father we will get a CT scan at our expense.”*

(Interview 7, male, 54 years old, rural, south WB)

*“For laboratory exams in Ramallah they told me that the biological treatment does not work with me, so we went to “A” hospital in Jerusalem and they took a biopsy to Israeli labs, and told me that one of the biological treatments works with me, so we came here (to this Palestinian hospital on the West Bank) and told them and I began treatment because the biological medication was here at this hospital on the West Bank. I took six sessions and then the medication was cut (no longer available), so they transferred me to the East Jerusalem hospital for the six remaining sessions, and I finished them and came back here.”*

(Interview 6, male, 45 years old, rural, south WB)

##### Oncology surgery

Some governmental hospitals offer this service, while referrals are provided for some specific procedures. However, the referral process is sometimes confusing to patients who face delays similar to referrals for diagnostic services. Delays in referral for surgery had a negative consequence on patients’ health, compounded by the complications of having a Jerusalem ID and living in the West Bank as this patient illustrates:

*“I took six chemotherapy sessions at “A” hospital, and went to the doctor and did a nuclear test and my situation was excellent…but was told I needed an operation… But because I have a Jerusalem ID, I had to go back and forth to obtain a referral to the Palestinian hospital, it took me time. And the situation became bad, the growth went around the pelvis and it made it difficult to have an operation……It is due to their neglect…”*

(Interview 3, female, 38 years old, urban, south WB)

A patient described the referral issues she encountered when undergoing head and neck surgery to remove a tumor. She finally went to a Palestinian government hospital and refused to leave until the surgery was completed because further delay would worsen her condition.

“*This doctor pushed me to that doctor, and that pushed me to that… they said there are no surgeons in the West Bank hospitals, your operation should be done at “A” hospital (in East Jerusalem). We went to Ramallah for a referral, and they told us that the doctor responsible for referrals is traveling… Either this is a game, or they making a mockery of people…”*

(Interview 19, female, 48 years old, rural, north WB)

In addition to the above surgery related difficulties, another patient reported on his experience when he had surgery in a governmental hospital to remove a tumor in the colon. He was asked to bring a device needed for the surgery at his expense. He was told that his surgery would have been postponed for three months if he could not bring the device. Following his colon surgery, he also needed a colostomy bag with its base in addition to needles, and he purchased them from private companies because the Palestinian Ministry of Health did not have them.

*“These bags are not available at the Ministry of Health and every day I need two… I can only find them from companies which sell medical equipment, and you buy the bag with the base for 35 ILS daily, and they do not cover any of the costs.”*

(Interview 21, male, 36 years old, rural, center WB)

Furthermore, some patients decided to seek surgical treatments in private hospitals because of referral delays or other problems such as physicians’ strikes or when they needed immediate medical intervention that could not wait.

*“I came and found that surgeons were on strike… so I did not want to wait, I did both operations at my expense….”*

(Interview 4, female, 47 years old, rural, south WB)

Other than the financial difficulties that the patients face in accessing health care, some interviewees especially those who do not live close to treatment centers elaborated on the transportation difficulties and financial burden related to reaching treatment centers:

“*Transportation, financially, is expensive and tiring. We are patients, we should have priority, we are cancer patients, and we should have transport and everything available to us.”*

(Interview 2, female, 59 years old, urban, south WB)

*“I come from a southern town with a private taxi, and they take 150 ILS every day…. To be honest, financially you cannot expect to have 400-500 ILS available to come and go, in addition to hospital expenses. You do not have that kind of money.”*

(Interview 7, male, 54 years old, rural, south WB)

##### Chemotherapy treatment

Interviewees stated that this treatment is generally available at oncology hospitals. Most participants reported that they were informed of the treatment plan in terms of the number of chemotherapy treatments.

Some participants stated that there is a lack of information provided to patients by health professionals regarding their treatment that also applied to the side effects of the chemotherapy treatment.

*“The chemotherapy I took made blisters in the mouth and dryness in the palms…. And slight nausea but the doctor did not tell me hair would fall….”*

(Interview 5, male, 33 years old, rural, south WB)

Patients also reported experiencing delays in beginning their chemotherapy sessions, and were bothered by the long waiting time they have to endure till they receive their treatment, and by being asked to come on another day.

*“When we were upstairs at the hospital, we would come at 8 am or before, and the medication would come to us at 3 pm or 4 pm… at 8 am they come and take tests, and your tests take about an hour to come out, so around 10 or 11 you should get your medications … but I do not get my medication until 3 or 4 pm… a whole day is lost.”*

(Interview 6, male, 45 years old, rural, south WB)

“*You do the tests and you wait and you stay a long time while you wait between the tests and the treatment.”*

(Interview 15, female, 30 years old, rural, north WB)

#### Service delivery and quality

##### Consultations

In addition to the variations of information shared by the oncologists, there were also differences in the consultation time given to the patients by oncologists. Some patients’ views were positive, feeling that oncologists work within their full capacities given the high number of patients. However, at the same time they suggested improvements related to the Palestinian Authority’s investment in health.

*“The government should take into consideration that one cannot do more than one’s capacity. They have a lack of doctors…. They should employ more people, but to have one doctor for an entire district like this district, who will he be able to see? Even the doctors are oppressed, they are right and they are wrong, but in the end, they are oppressed…”*

(Interview 7, male, 54 years old, rural, south WB)

Other participants complained about the available time for interaction with the oncologists. This prompted some patients to seek consultations with oncologists at their private clinics due to the limited time available for them at the hospital.

*“with doctors it is difficult, if you want to talk to them, you need wasta [connections], they do not answer…if you go to the doctor’s private clinic he answers you… here at the governmental hospital, he cannot see you at all, too busy…. if you want to talk to the doctor, you need to go to the clinic and pay 100 ILS, he would sit with you for an hour and laugh with you….”*

(Interview 11, male, 58 years old, rural, north WB)

With regard to nurses, most participants expressed their appreciation for the nurses, particularly those who work in daycare treatment, acknowledging that the nurses are overworked. Nevertheless, less positive feedback was reported by some participants for the nurses responsible for inpatient wards.

*“Some nurses are the sweetest you like to see them 24 hours a day, some nurses you do not accept a glance from them, not all of them… only 1% of them… By God this night no one came to us, no one, even when the IV drip was finished.”*

(Interview 2, female, 59 years old, urban, south WB)

##### Infrastructure

Complaints about the lack of system capacity also extend to the infrastructure of the treatment facilities, especially in the inpatient ward of the oncology hospitals. One of our oncologist interviewees confirmed that the inpatient ward in the hospital he works at has only 20 beds and is overloaded. He noted that they sometimes admit cancer patients to other wards. As we see below, some patients reported delays in being admitted to the rooms and waiting for a bed to become available.

“*I come and I sit on a chair for a long time before they find a place for me in the inpatient ward.”*

(Interview 2, female, 59 years old, urban, south WB)

*“The size of the patient-load (cases) is large… meaning that when I come to register at the hospital, I wait in line for a bed to be empty or for space. I was supposed to sleep at the hospital on Thursday, but it was not possible because there was no space, so we postponed until Sunday.”*

(Interview 10, male, 48 years old, urban, south WB)

Most participants also reported lack of cleanliness in the rooms such as dusty surfaces, un-sanitized equipment, and dirty bed sheets. They mentioned that the rooms are overcrowded with patients beyond their capacity, toilets are shared, some beds are broken, blankets and pillows are insufficient, and there is no space for accompanying family to sleep. Some were bothered by the noisiness of visitors and the lack of privacy.

*“I get upset because of the lack of cleanliness, because of the general services… we ask and nothing happens. This is how the floor is[pointing to the floor], no cleanliness, they do not wipe the floor. Here there is no cleanliness, and we (the patients) clean.”*

(Interview 2, female, 59 years old, urban, south WB)

*“The oncology section on the third floor, I do not know if you have been there or saw it…. Terrible… rooms for two have three patients other than companions… pressured, no privacy, cleanliness is almost non-existent… there is no space for companions to sleep…. they sleep on the floor if they obtain a cover… sometimes there is no pillow for you (the patient), not for your family (accompanying you).”*

(Interview 6, male, 45 years old, rural, south WB)

##### Timeliness and integration

Fragmentation of services was a common concern. For example, patients demanded easy access to receive prescribed medications and other treatment-related services such as blood testing given that it is difficult for them to go to multiple centers for different health care needs.

*“Most of my treatment is here in this building, and the blood tests are in another building…. For sure it would be more comfortable for one if they were in one place instead of going up and down, going and coming… this is tiring.”*

(Interview 20, female, 57 years old, rural, center WB)

##### Drug availability, pain and palliative care

In general, nearly all interviewees reported that the unavailability of medications at the Palestinian oncology hospitals is a significant difficulty. Painkillers, laxatives, vitamins, and gastrointestinal medications were among the reported missing medications. Therefore, the patients pay for these medications out of pocket, which create additional financial burdens for many patients and their families.

*“Pain killers I buy from my own pocket, they are found in the market, but not available with the governmental insurance. I need stomach medications because what they give me for my stomach is not as effective as other medications which I buy at my personal expense outside the hospital….”*

(Interview 6, male, 45 years old, rural, south WB)

Patients in pain were the ones who emphasized the shortage of painkillers the most, indicating that they get inconsistent supplies of painkillers medications. Furthermore, some noted that paracetamol is usually administered to treat their intolerable pain, which they found to be inadequate.

*“Pain is terrible … there is a pain killer which is close to morphine, a strong pain killer that I used to take in the East Jerusalem hospital, but it is not available here of course…I do not know if they just did not give it to me, they gave me this medication but it did not work with me.”*

(Interview 6, male, 45 years old, rural, south WB)

*“A painkiller, paracord that needs a prescription. Yes, the doctor wants to give it to me to use when necessary. He tells me to take one pill and if needed to take two pills…first, I took paracord and benefited from it and then I stopped benefiting… they (at the hospital) give me the new medication (Paracetamol 325). Maybe the two first hours the pain goes and then it doesn’t work.”*

(Interview 12, male, 57 years old, urban, north WB)

*“I’m in a lot of pain. Every day a certain pain is not the same pain from the first day to the last day…. The doctor wrote Panadol and told me that I can take it whenever I need. I sometimes take 4 or 6 pills a day and there is a pain that remains. I told the doctor and he told me that this is normal, and it is a period and that I have to endure it.”*

(Interview 15, female, 30 years old, rural, north WB)

The experiences of patients reveal that there is a systemic problem with pain management. There are inconsistencies in the availability of medication, in prescribing practices, and in the training of the health staff. One of the oncologists we interviewed confirmed the medication issue. He noted that despite the importance of appropriate pain killers to the quality of life of patients, most painkillers medications are not available and those that are available are in low supply.

*“The problem is that cancer patients are treated as if they will die and everyone forgets about them. They forget that these patients are in pain regardless of their disease.”*

(Oncologist, male, south West Bank hospital)

Moreover, palliative care is seen as “missing” in cancer care in the West Bank.

*“There are no clear guidelines or protocols with cancer care generally, and palliative care specifically at the MOH. There is no clear structure for palliative care, nor is there a dedicated team. There is a need for home visits, especially with the end-of-life patients and psychosocial support but there is currently no structure for it within the current system.”*

(Oncologist, male, center West Bank hospital)

In line with this, another oncologist said:

*“There is no palliative care team or department in our hospital. Emotional and psychosocial care is not available: the oncologist does the job of talking and communicating with the patient and their family.”*

(Oncologist, male, south West Bank hospital)

##### Psychological support

The need for professional psychological support was deemed very important by most interviewees, even though they acknowledged receiving support from their families. Some of the reported psychological impacts due to the illness include nervousness, bad temper, feeling incapacitated, and overthinking. Many patients highlighted the importance of high morale and good psychological wellbeing as a key part of their treatment.

*“I wish they would sit with me and tell me what is happening… or what you can do… such things are a must, but here, none.”*(Interview 20, female, 57 years old, rural, center WB)

As the quote above shows, many patients noted that this aspect of care was largely missing. While many would say that they are in good spirits in general, when we asked about their care needs, they would frequently mention the need for psychological support.

## Discussion

This study describes the difficulties that patients with advanced-stage cancer experience in accessing oncology services in the West Bank from the point of seeking a diagnosis. The findings reveal that cancer patients encounter multiple barriers in accessing oncology services throughout their treatment journey, underlining difficulties Palestinians in general face in accessing health care (16–18). Some of these challenges are shared with other conflict-affected areas (5, 7), while others are specifically related to prolonged Israeli military occupation and its effects on the capacity of the Palestinian health system to deliver comprehensive cancer care.

One of the significant challenges encountered by cancer patients is the unpredictable, lengthy process of obtaining Israeli permits to access cancer care in East-Jerusalem not available on the West Bank. The delays or refusal are consistently confirmed in World Health Organization reports for patients seeking Israeli permits, including cancer patients (20, 22). The refusal or delay of Israeli permits results in delays in diagnosis and treatment, negatively impacting health outcomes of cancer patients (17, 20). Additionally, interruption of cancer treatment, related to delays from the Israeli side in providing chemotherapy-related drugs and diagnostic materials are major blockages in the cancer pathway. Interruption of chemotherapy treatment is an ongoing challenges for cancer care in the oPt (17, 19) and experienced in other conflict-affected areas (7).

Cancer patients face challenges in accessing advanced diagnostic services such as nuclear scans, CT scans, and MRIs, which are unavailable at governmental hospitals, not functioning, or inaccurate in some cases. Earlier studies were in line with these findings which confirmed that nuclear medicine and PET scans are not available at Palestinian governmental hospitals and patients are referred to East Jerusalem or Israel to obtain such services (12, 13, 19).

Moreover, our study showed that cancer patients face barriers in accessing surgical oncology services. These barriers included a lack of guidance to the patients on where to seek their surgeries. This sometimes leads patients to go to multiple hospitals in search of the needed surgery as a result of not being provided by the appropriate referral. Our finding is consistent with earlier research results stating that the oPt lacks specialized centers in oncology surgery, and the existing surgery services are not well-structured (13).

Additional referral barriers in accessing diagnostic and surgery services were also reported by the participants. Problems included delays in getting referral approval from the Palestinian Ministry of Health which negatively affected the health condition of some patients. Timely approval for cancer care referrals is a persisting challenge (17, 25, 26). While some participants waited for the referral approval, others were forced to receive treatment at their own expense which added a financial burden on the patients and their families. Out-of-pocket costs for cancer patients and their caregivers in low and middle-income countries account for an amount that equals 42 % of their annual income (27).

Another significant issue is the consistent shortage of medications at the governmental hospitals that patients are required to take home for their treatment. This is a persistent issue (12, 28). Specifically, the lack of pain medications highlights the absence of a palliative care program in cancer care, confirmed by the oncologists participating in the study. Palliative care is an essential health service within Universal Health Coverage (29) and is cost effective (30). Yet, people with advanced cancer in conflict- affected areas are especially in need of palliative care as they report compounded trauma, that of living in wars and conflicts and that of enduring the symptoms and treatments of their cancers at the same time (29, 31). Indeed, it is concerning to note the absence of palliative care and the distress due to uncontrolled pain among cancer patients in the West Bank.

Concerning other deficiencies in health services, the oncologists’ lack of communication and limited consultation time with patients was one of the important problems noted by patients. In accordance with our findings, an Indian study found that almost half of the studied cancer patients required information related to their treatment without being able to receive such information (32).

The poor infrastructure of the treatment facilities was also among the cancer patients’ concerns. Prior studies have noted that health system challenges for cancer patients might be partially explained by the Israeli military occupation and economic crisis of the Palestinian Authority in financing the health care system in general, including cancer care (19).

In summary, barriers to accessing cancer care in the West Bank are structural and due to the constraints imposed by the Israeli military occupation, while some are related to the Palestinian health system’s shortage of resources which relate to living under Israeli occupation as well as failures in adequate management of the healthcare systems. Patients called for significant improvements in services, including comprehensive diagnostic services; obtaining timely referrals to services; and providing all oncology pathway services in the same place. Other recommendations included increasing numbers of health staff (especially oncologists and nurses); having a consistent supply of medications; expanding inpatient wards and making improvements in infrastructure and cleanliness.

Finally, cancer patients accessing care in chronic conflict-affected settings (such as is the case of the oPt) will continue to suffer if the root causes of these access constraints persist. Palestinians need to obtain their right to health, which means that in the absence of freedom, justice and a fair political solution to the Palestine-Israeli conflict, a fragmented and underdeveloped health system will not be able to sustain cancer services nor guarantee their adequacy.

## Data availability statement

The datasets presented in this article are not readily available. Requests to access the datasets should be directed to SM, smitwalli@birzeit.edu.

## Ethics statement

Ethical approval was granted by the Research Ethics Committee at the Institute of Community and Public Health, Birzeit University (ref number 2020(3 – 1) and at Kings College London (ref number HR-20/21-18199). Written informed consent for participation was not required for this study in accordance with the national legislation and the institutional requirements.

## Author contributions

RG, WH, and SM conceptualized the research idea, and designed the study. RH read and commented on the draft paper. SM conducted the field work, analyzed transcripts and wrote the draft paper, and finalized the article. WH participated in the field work and writing of the draft paper. All authors contributed to the article and approved the submitted version.
